# When to Advise Operation in General Practice

**Published:** 1916-12

**Authors:** 


**When to Advise Operation in General Practice.** By A. Rendle Short, M.D., B.S., B.Sc. Lond. Pp. vi., 279. Bristol : John Wright & Sons Ltd. 1916. Price 5s. net.

This handy book largely consists of the application of the knowledge so ably collected and edited by Mr. Short in the *Index of Prognosis and End-Results of Treatment.*

Patients cannot be treated like mathematical propositions, and there is in each case an individual factor which must be considered, and which prevents the universal application of hard and fast rules as regards the desirability or otherwise of operation.

The author recognises these limitations, and tells us that "we can only legislate for the normal."

The knowledge of what is usually necessary in the average case is most helpful to the general practitioner, and the opinions of the author are expressed in clear language, which is summarised in dogmatic form at the end of each chapter.

There is a creditable vein of conservation in the book, which may help to dispel the popular illusion that the consulting surgeon always wants to "use the knife."

The practitioners for whom this book is intended may rely upon its sensible advice, and will find it of great value in cases of doubt and difficulty.

There is a good index, which further increases the utility of the work.

"Alinement" occurs twice on page 226.

